# Clinical case of Botryomycome fulminant at the Center of Diagnostic and Treatment of Tuberculosis of Baleng (West - Cameroon)

**DOI:** 10.11604/pamj.2013.14.131.2387

**Published:** 2013-04-04

**Authors:** Michel Noubom, Bruno Kenfack, Jean Hubert Donfack, Fabrice Djouma Nembot, Zacharie Sando

**Affiliations:** 1 Department of Biomedical Sciences, Faculty of Science, P.O. Box 067 University of Dschang, Cameroon; 2 Center of Diagnostic and Traitement of Tuberculosis of Baleng, P.O. Box 375 Bafoussam, Cameroon; 3 Faculty of Medicine and Biomedical Sciences, P.O. Box 1364 University of Yaoundé I, Cameroon

**Keywords:** Inflammation, pyogenic granuloma or botyomycome, Botryomycome fulminant, diagnosis, Dapsone, Rifampicin

## Abstract

Botryomycome also called pyogenic granuloma, is an inflammatory tumor of the skin and mucous membranes often caused by superinfection of minor traumatism. Its uniqueness lies in its granulomatous organization in which each granulation contains bacteria and the predominance of many newly formed blood vessels, with the lights on variables ratings and a turgid endothelium responsible for the ulcero-hemorragic appearance. This delicate condition poses a real problem of treatment which included: an appropriate antibiotherapy, surgical removal or electrocoagulation which, until today is more difficult in countries with limited income. An octagenarian came to our hospital with a large mass on the left foot. This mass had the appearance of a malignant tumor. However, the anatomopathologic diagnosis showed that it was a pyogenic granuloma. The importance of the mass, its location on the sole of the foot, imposed the functional impairment of this member. Electrocoagulation is not available in our hospital and the surgical excision proposed was refused by the patient. The patient was treated with the antibiotics Ceftriaxone and Gentalline at indicated doses for 15 days. After the failure of that antibiotherapy treatment, as a last resort, the patient was treated for a month with another combination of antibiotics (free of cost) made of dapsone and rifampicin. This new combination gave very good results. This example shows dapsone and rifampicin can be use as a new weapon for the management of pyogenic granuloma in countries with limited incomes.

## Introduction

Botriomycome or pyogenic granuloma is a benign vascular inflammatory tumor of skin or mucous membrane, often secondary to minimal trauma. It looks like a small shiny erythematosus nodule, painless, which grows gradually in one to three weeks to reach 0.5 to 2 cm in diameter. It may be sessile or pedunculated, a narrower neck is often observed at the base. It is sometimes epidermised, eroded, crusty and black [1]. It is a benign tumor of vascular origin in the superficial dermis, but hypodermic forms have been reported [2]. Clinically, it looks like a pseudo tumoral ulcero-hemorragic budding with patches of suppuration. It is a vascular tumor of variable size, soft fleshy and often benign inflammatory burgeoning, bleed easily on contact, bright red. Botriomycome typically appears after a small wound [3]. In most cases, it is the result of staphylococcal infection of the subcutaneous tissue in its chronic manifestations. Although they are found in adults, it is the disease of childhood [4]. Predilection sites are acrales areas such as fingers and toes and they often accompany ingrown toenail [5]. On the histological plan, the main mass is consists of many newly formed blood vessels with variable sizes and turgid endothelium. This set is included in a loose stroma and edematous. It is characterized by a granulomatous organization in which each granulation contains bacteria [5]. The treatment is based on prolonged antibiotic therapy, electrocoagulation or cryotherapy (in the case of the availability of equipments). Given the “barrier effect” of granulomas, surgical excision may be proposed if the mass is too large.

## Patient and observation

Mr. X, 80 years old farmer came to the consultation for the following signs: exertional dyspnea, chronic ulcerated and a burgeoning wound on the left foot, bleeding on contact for more than three months.

The disease started about three months prior to consultation and was marked by painful tumefaction, swelling and warmth of the sole of the foot, followed by desquamation and budding. The patient spent several weeks in the District Hospital of Batcham (West-Cameroon) and was put under cloxacillin at a dose of 2g per day for two months without success. The budding taking over the entire sole of the foot, the patient decided to consult the Center of Diagnostic and Treatment (CDT) of Tuberculosis of Baleng which, in addition to being the largest CDT in the West region of Cameroon, is also the center for detection and treatment of leprosy. Thus, the orientation of the patient in our hospital was motivated by a suspicion of leprosy. The family history of the patient was unremarkable.

The personal history of the patient showed that he was hypertensive with known heart failure for 05 years. He was under cardiurine (captopril 50mg / HCT 25mg) at a dose of one tablet daily and digoxin 0.25 mg at the dose of one tablet every 12 hours. Moreover he had a lipomectomy in 2009. Nothing else was remarkable. The physical examination of the patients revealed an impotence of the left lower limb and a painful wound on the sole of the foot on the same side. Overall, he is an octogenarian with an aspect of undernutrition and slight muscle lost. He presents a moderate hypertension (170/100 mm Hg) and a cardiac arrhythmia. The ulceration of the left foot was important and burgeoning, with few hemorrhagic foci and bleeding on contact. The diagnosis of malignancy with superinfection of the sole of the left foot in a hypertensive patient was made.

### Blood workouk

FBC: WBC: 5,5 x 10^3^ /µl; RBC: 4,58 X 10^6^ / µl; HGB:12.9 g/dL; MCV: 80 Fl MCH: 29 Pg; MCHC: 35.3g/dL; Plt: 275 x 10^3^ /µl; fasting blood glucose: 0.89 G/L; urea: 0.42 G/L; creatinin: 6.10 mg/l; HIV serology was negative; test of leprosy on the lesion was negative.

**Figure 1:**
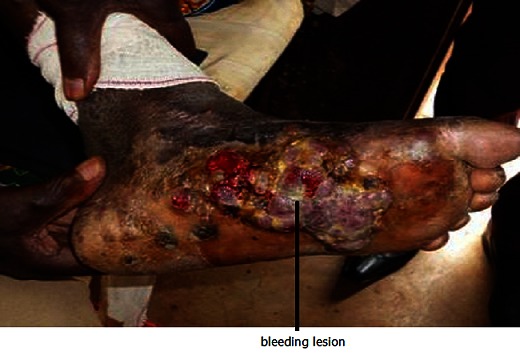
Photograph of the patient's foot at the entrance to the Hospital; case of pyogenic granuloma (by Dr. Noubom).

Culture of the pus collected from the foot revealed the presence of *Staphylococcus aureus* (sensitive to cefixime, aminoside, amoxicillin and clavulanic acid, rifampicin). A biopsy was done on three different location of the wound. Microscopically, the sample showed a lesion that was focally ulcerated on the surface and superficial dermis. There was a lesion made of granulation tissue with elongated or oval vascular structures. These structures were made of non atypical benign endothelial with a mixed inflammatory infiltrate rich in fibroblasts. There was also a significant fibroblastique proliferation made up of elongated fusiform cells. On the surface, the flaps of malpighien epithelium were slightly hyperplastic: no malignancy. As a conclusion, it was a case of pyogenic granuloma also called botryomycome (Figure 1)

### Treatment

After the culture result indicating the presence of *Staphylococcus aureus*, and anatomopathologic analysis of biopsy suggestive of pyogenic granuloma, the patient's family refuse the surgical excision. The patient was then treated with ceftriaxone 1G X 2 per day and gentalline 80mg x 2 per day for 10 days, in addition to the daily treatment of wound after whashing with water containing betadine. The evolution of the patient was not very satisfactory, and surgical excision proposed again to the family, was again rejected. Moreover, taking in consideration the pharmacodynamics action of rifampicin and the effect of dapsone on the chronic leprous lesions, the following treatment was proposed to our patient: dapsone and rifampicin which were available free of cost in our Center. Our patient was then submitted to dapsone/rifampicin for a period of one month of treatment, the appointment was given at the end of the first wafer. After a month, the response was very encouraging with rather remarkable regression of the swelling and the total disappearance of exuding wounds which bled at the slightest touch.

## Discussion

By seeing this tumor on this patient, the first impression was to think to Kaposi which is among the differential diagnoses of pyogenic granuloma. Other diagnoses such as angioma tuft, hemangioma, lobular angioma, micro capillary angioma, Masson tumors, reactional angioendotheliomatosis, angiokeratoma and bacillary angiomatosis [6, 7] could draw our attention in front of this kind of image. These observations prompt us to think directly to bacterial culture and histological examination as key for the diagnosis of this tumor. Furthermore, an anatomopathologic analysis may also eliminate achromic melanoma which could also a concern in adults [1].

In adults, botyomycome is often due to trauma, sometimes surgery; it can also occur during pregnancy, especially on the gums and is favored by some systemic treatments such as isotretinoin and protease inhibitors [1]. In the case of our patient, apart from the fact of his age, none of these favoring factors was advanced in the history of the disease.

**Figure 2:**
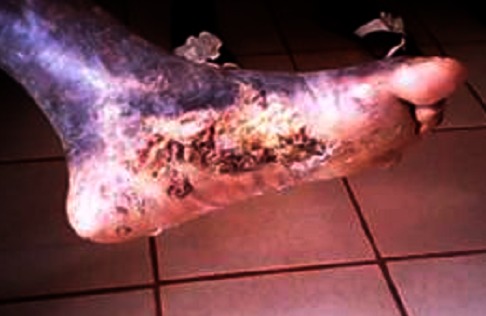
Photograph of the patient's wound after one month of treatment (by Dr. Noubom).

The treatment of choice is surgical removal under local anesthesia and if necessary, by electrocoagulation [1, 8]. This therapeutic decision is often subjected to numerous relapses [1]. It is by taking into consideration existing treatment protocoles and the refusal of the patient for surgery, that we tried the combination Dapsone-Rifampicin. As shown in Figure 2, this combination provided spectacular results; it is a combination of very broad spectrum antibiotics with good tissue diffusion [9]. The anti-inflammatory and immunomodulatory activities of antibiotics may play an important role in the effectiveness of this treatment [10]. Some intracellular antibiotics, particularly macrolides, are endowed with anti-inflammatory and immunomodulatory properties [11, 12, 13]. Rifampicin is well known as intracellular antibiotic.

## Conclusion

This clinical case summarizes challenges in the diagnosis of pyogenic granuloma or botyomycome that suspiciously looks like a malignant tumor, and presents the possibility of treatment of this disease with the combination of rifampicin and dapsone. Thus, for countries with limited income, this therapeutic combination could be a new focus for the management of fulminant pyogenic granuloma.
